# Advancing cancer immunotherapy: a vision for the field

**DOI:** 10.1186/s13073-019-0662-6

**Published:** 2019-07-29

**Authors:** Noel F. C. C. de Miranda, Zlatko Trajanoski

**Affiliations:** 1 Department of Pathology, Leiden University Medical Centre, Albinusdreef, 2333 ZA Leiden, The Netherlands; 2 Biocenter, Institute of Bioinformatics, Medical University of Innsbruck, Innrain, 6020 Innsbruck, Austria

The advent of T cell checkpoint blockade immunotherapy has changed the paradigm of oncologic treatment for several cancers following decades during which the field had fallen short in delivering effective cancer therapies. The triggering and rescuing of anti-tumor T cell activity through the targeting of T cell checkpoints has led to durable clinical responses, including curative outcomes, in patients diagnosed with extremely challenging pathologies such as advanced non-small-cell lung cancers, melanomas, renal cell carcinoma, and bladder cancers. Fittingly, the 2018 Nobel Prize in Physiology or Medicine was awarded to James P. Allison and Tasuku Honjo for the discovery of inhibitory mechanisms in T cells that are mediated by the CTLA-4 and PD-1 checkpoints, which are currently prime targets of immunotherapeutic antibodies. In order to deliver on the promise of cancer immunotherapy, preclinical and clinical studies, as well as efforts by pharmaceutical companies, have skyrocketed in the past few years. While we are learning a great deal about the biology underlying treatment responses, the fraction of patients that benefit from immunotherapy is still rather small: it was estimated that the percentage of advanced and metastatic cancer patients who responded to checkpoint blockade therapies in 2018 was 12.46% [1], which superior to the performance achieved by oncologic treatments that target specific genomic alterations in tumors (such as kinase inhibitors).

In contrast to conventional targeted therapies that have generally focused on interfering with cancer cells, checkpoint blockade immunotherapies exert their effects (directly and indirectly) on several immune cell subsets, including effector and regulatory T cells and myeloid cells. Their performance is also influenced by features of the tumor cells as well as by other components of the tumor microenvironment [2, 3]. Furthermore, checkpoint blockade acts at distinct anatomical locations in patients: T cell priming and activation occur primarily at lymph nodes and T cells circulate through the blood stream to reach the tumors [4]. The complexity of the two ecosystems at play—the tumor microenvironment and the systemic immune system—requires a comprehensive and systematic analysis of cellular and animal models as well as patient samples, using a toolbox of molecular and computational tools that capture the biological complexity of these systems and which factor in spatial and temporal dynamics. Classical models such as cell lines, transplantable mouse models, or patient-derived xenografts have major limitations in the context of cancer immunotherapy, and innovative models including tumor organoids co-cultured with immune cells, humanized mice, or human tumor explants are increasingly used to dissect tumor-immune cell interactions [5, 6]. Importantly, it is crucial that clinical trials for cancer immunotherapies are coupled to research endeavors that include a comprehensive study of pre-treatment and post-treatment samples, which will reveal the mechanisms of primary and acquired resistance to immunotherapy and identify biomarkers that are able to predict treatment response [7, 8].

Two of the major challenges in the field are the need to improve the stratification and selection of cancer patients who are likely to respond to state-of-the-art immunotherapies and the development of innovative approaches that extend the benefits of cancer immunotherapy to a greater proportion of patients [9]. To address the former problem, cutting-edge technologies, including bulk and single-cell RNA sequencing as well as multidimensional immunophenotyping approaches (such as flow and mass cytometry), are being employed to decipher the complexity and the heterocellular crosstalk in the tumor microenvironment and to investigate systemic cellular crosstalk [10, 11]. An additional level of complexity is provided by the demonstration that an individual’s (gut) microbiome strongly influences their host immunity and, consequently, their response to cancer immunotherapies [12].

Another open challenge in the field is the identification of antigens that elicit T cell responses in patients, particularly those derived from cancer somatic mutations, which are known as neoantigens [13]. While the determination of neoantigens from next-generation sequencing data and algorithms that predict the binding affinity of mutated peptides to human leukocyte antigen (HLA) molecules are constantly improving, a method that accurately determines peptide immunogenicity by correctly weighing different features (for example, expression levels, stability, HLA-binding affinity, and so on) but which also integrates the T cell receptor (TCR) repertoire of patients is still lacking. To achieve this, it will be necessary to expand the currently available data on the detection of neoantigen-reactive T cells in cancer, which should include the targeted epitopes and the TCR sequences of neoantigen-reactive T cells [14]. Further, the field has begun to look beyond the canonical coding genome for the discovery of cancer-specific antigens because oncogenic events are likely to result in the creation of novel open reading frames and in the expression of alternative transcripts [15]. Finally, beyond CTLA-4 and the PD-1/PD-L1 axis, several immunotherapeutic targets mediate co-inhibitory immune checkpoints (such as LAG-3 or TIM-3) or co-stimulatory immune checkpoints (such as OX40 and GITR). These targets are currently being explored in clinical trials [16], which demand the identification of a mechanistic rationale for combination therapies that also include other treatment modalities (such as chemotherapy, radiotherapy, or kinase inhibition). Given the large number of clinical trials that involve combination therapies (as of September 2018, more than 2200) [17]), it is questionable how sound the evidence will be because a large number of patients are involved, many specific combinations may be used, and the risks for adverse effects resulting from the combination of several drugs must be predicted.

In this special issue of *Genome Medicine* original research, reviews, and opinions from the leaders in the field address these challenges and provide novel insights, as well as information on technological and analytical advances, to support cancer immunology research. In a multi-omic study of hepatocellular carcinomas by Löffler et al. [18], exome-derived mutated HLA ligands were found to be very rare, suggesting the need to broaden tumor-antigen discovery efforts for malignancies with low mutational burden. Such work also raises important questions about the accuracy of existing approaches for neoantigen identification, as discussed by Ehx and Perreault [19]. Finotello et al. [20] present a new tool, quanTIseq, that supports the study of the tumor microenvironment, specifically the characterization of the immune contexture—that is, the type, density, and functional orientation of tumor-infiltrating immune cells—using bulk RNA-seq data. These authors demonstrate quanTIseq’s utility in the context of combination therapy and immune-related adverse events, and show how it may shed light on the immune-cell types that underlie differential patients’ responses. In an Opinion article, Lhuillier et al. [21] describe a model in which radiation therapy can expose immunogenic mutations to the immune system, which has the potential to predict which patients may benefit from treatment with combinations of radiotherapy and check-point blockade therapy.

From the studies in this special issue and from a plethora of other published work, it is clear that the use of multiomic technologies to assess exomes, epigenomes, and transcriptomes has profound limitations, and that it will be necessary to characterize the tumor microenvironment comprehensively using multimodal assessments. Such work will employ medium-throughput technologies that include spatial information, such as multiplexed immunofluorescence, imaging mass cytometry, or in situ RNA-detection platforms [22]. Furthermore, the modeling of the biological complexity observed in tumors remains a huge challenge in the field. Will scientists be able to accommodate the various components of the tumor microenvironment in in vitro models such as organoids? Or are short-lived tumor explants best suited to preserve the multicellular context of anti-tumor immune responses? Both approaches lack an essential component of in vivo anti-tumor immune responses—the systemic immune system which, for now, can only be accounted for with animal models.

We strongly advocate that the integration of clinical, molecular, and cellular data, as well as the sharing of those data across the community, is of utmost importance in identifying biologically relevant signals that are buried in experimental noise [23]. It will be necessary to develop and maintain dedicated repositories for the data generated in clinical trials and functional experiments, which catalog not only raw molecular data (to enable re-analysis using improved tools) but also the associated meta-data, including pre-treatment information, outcome, and adverse effects (to enable correlative analysis). As a first step, it will be necessary to define a community consensus on the minimal information about a cancer immunity (MIACI). Besides technical issues such as the amount of the data generated and the required storage capacity, ethical and data protection issues need to be addressed in order to make the data available to the community. Moreover, innovative approaches are required to measure a multitude of parameters in a single sample, the size of which is often limited when derived from patients. Ideally, the maximum number of dimensions should be captured from a single sample, including therapeutically actionable genomic alterations in tumors, bulk transcriptional profiles, and deep immunophenotyping (including a comprehensive identification of immune cell subsets, their functional state, and their tissue location in relation to tumor cells). The development of such technologies is now taking off and it is expected that, in the coming years, these procedures will become standard methodologies in cancer research [24, 25].

In conclusion, as the number of patients being treated with cancer immunotherapy increases in the coming years, it is expected that our field of research will make another tremendous leap towards understanding the intricate mechanisms that are at play in cancer immunity, and which ultimately influence a patient’s response to immunotherapy. That knowledge, in combination with technological advances that support not only the full characterization of biological samples but also the high-throughput production of therapeutic solutions (such as neoantigen vaccines, engineered T cells), will result in the development of novel immunotherapeutic approaches that will broaden the benefits of cancer immunotherapy to a larger number of cancer patients.

## Abbreviations

HLA: Human leukocyte antigen
TCR: T cell receptor
